# Subclinical Cardiac Diseases and the Role of Extracellular Vesicles in Patients with Hemophilia A Treated on-Demand

**DOI:** 10.1177/10760296261432444

**Published:** 2026-03-09

**Authors:** Yanan Zong, Maren Maanja, Todd T. Schlegel, Martin Ugander, Jovan Antovic, Apostolos Taxiarchis, Roza Chaireti, Xiangdong Kong

**Affiliations:** 1 Coagulation Research, Molecular Medicine and Surgery, 27106 Karolinska Institutet, Stockholm, Sweden; 2 Department of Clinical Physiology, 59562 Karolinska University Hospital, Stockholm, Sweden; 3 Department of Clinical Physiology, 27106 Karolinska Institutet, Stockholm, Sweden; 4 Nicollier-Schlegel SARL, Trelex, Switzerland; 5 Kolling Institute, 60086 Royal North Shore Hospital, and University of Sydney, Sydney, Australia; 6 Clinical Chemistry, Karolinska University Laboratory, Medical Diagnostics Karolinska, Karolinska University Hospital, Stockholm, Sweden 59562; 7 Department of Hematology, 59562 Karolinska University Hospital, Stockholm, Sweden; 8 Department of Medicine, Solna, 27106 Karolinska Institutet, Stockholm, Sweden; 9 The Genetics and Prenatal Diagnosis Center, The Department of Obstetrics and Gynecology, 191599 The First Affiliated Hospital of Zhengzhou University, Zhengzhou, China

**Keywords:** hemophilia A, coronary artery disease, extracellular vesicles, global hemostasis, advanced electrocardiography

## Abstract

**Introduction:**

Patients with hemophilia A (HA) can reach normal life expectancy and suffer from coronary artery disease (CAD). The study evaluates the likelihood of subclinical CAD evaluated by advanced electrocardiography (A-ECG), global hemostasis and extracellular vesicles (EVs) in Chinese patients with HA treated on-demand.

**Materials and Methods:**

Patients with HA (n = 42) and age-matched male controls (n = 37) were included. The likelihood of having CAD was evaluated by A-ECG. Fibrin formation and fibrinolysis were assessed by the overall hemostatic potential (OHP) assay. EVs derived from platelets (PEVs), endothelial cells (EEVs) and leukocytes (LEVs), and expressing phosphatidylserine (PS + EVs), tissue factor (TF + EVs) or P-selectin (CD62P + EVs) were measured by flow cytometry.

**Results:**

The likelihood of CAD evaluated by A-ECG did not differ between patients and controls. Fibrin formation and clot stability were significantly impaired in patients. Patients had higher PEVs and CD62P + EVs. CD62P + EVs counts were inversely correlated with OHP, velocity and clot lysis time. Subclinical CAD did not correlate with OHP or EVs.

**Conclusion:**

Controls and patients with HA treated on-demand exhibited no differences in A-ECG, but varied in fibrin formation and clot stability. Larger studies are required to explore the significance of this finding in the context of CAD risk in this population.

## Introduction

The major improvements in the management of hemophilia A (HA) in recent decades,^1–4^ have prolonged life expectancy in those patients.^5^ This has led to several health challenges in this aging population, such as the occurrence of cardiovascular diseases (CVD), including coronary artery disease (CAD).^5,6^ Patients with HA have been shown to have lower mortality secondary to ischemic heart disease compared to the general population, probably due to impaired occlusive thrombi formation following the rupture of atherosclerotic plaques.^4,7^ Additionally, in patients with severe HA, the non-fatal atherothrombotic events appear to be less frequent compared to controls.^8,9^ This could imply that severe factor VIII (FVIII) deficiency might be beneficial against CAD and therefore that treatment with FVIII concentrate prophylactically or on-demand can theoretically attenuate this protective effect.

Extracellular vesicles (EVs) are nano-sized membrane vesicles shed from cells under normal or pathological conditions.^10^ EVs are potential diagnostic and prognostic biomarkers in CVD, including CAD.^11^ In patients with HA, the levels of phosphatidylserine (PS) positive EVs (PS + EVs) and platelet-derived EVs (PEVs) are inversely correlated with the global hemostatic parameters.^12^ However, the relationship between EVs and potential CAD risks in the elder HA patients has not been studied.

Different methods of advanced electrocardiography (A-ECG) measures have been utilized as an inexpensive and quick means to evaluate and detect cardiac disease with greater sensitivity compared to the standard ECG^13–16^ since various heart diseases, such as CAD and left ventricular systolic dysfunction have been shown to exhibit abnormal results on A-ECG^13,17,18^ The results of A-ECG can be incorporated to significantly improve the accuracy of diagnostic and prognostic evaluation for several disease conditions, including CAD, as well as heart age compared to conventional ECG.^13,17–20^

In this study, we evaluated risk factors for subclinical CAD as exhibited by an advanced electrocardiography (A-ECG) technique in patients with HA treated on-demand as well as male controls older than 30 years. We also studied global hemostatic methods and EVs levels, and their associations to the risk of subclinical cardiac disease. The hypothesis of the study is that patients with HA treated with FVIII concentrate on-demand might exhibit lower CAD risk, as evaluated by A-ECG, compared to controls due to a theoretical protective effect secondary to deficiency of FVIII.

## Patients and Methods

### Study Population

Male patients ≥ 30 years with congenital HA regardless of severity degree and treated with factor concentrates on-demand at the Center of Genetics and Prenatal diagnosis of the First Affiliated Hospital of Zhengzhou University were eligible for inclusion. An equal number of age-matched (≥ 30 years) male controls were recruited by placing a local advertisement. The study was approved by the Ethical Committee of the First Affiliated Hospital of Zhengzhou University. Written informed consent was obtained from all participants, and the study was performed according to the Declaration of Helsinki.

### Blood Sampling and Laboratory Analysis

Fasting peripheral venous blood was collected into 4 × 3 mL tubes with 0.129 M sodium citrate for coagulation assays and EVs analysis, and 1 × 3 mL serum-separating tube for the measurement of blood glucose and lipids levels. For the overall hemostatic potential (OHP) assay and EVs analysis, platelet-free plasma was obtained by double centrifugation of the blood sample at 3000 g for 10 min, at room temperature. The platelet-free plasma was aliquoted and stored at −80 °C awaiting analysis.

Serum glucose and lipids levels were analyzed at the local Clinical Chemistry routine laboratory. FVIII activity was measured according to the one-stage clot-based assay, and FVIII inhibitor levels by the Bethesda assay for all patients.

### Clinical Data Collection and Definitions

The following clinical data was collected: age at diagnosis and first bleeding, frequency of bleeding, frequency of on-demand treatment, hemophilic arthropathy, infection (hepatitis C virus (HCV) and human immunodeficiency virus (HIV)) and family history of HA.

Additionally, data on risk factors for CAD was retrieved from the medical records of both patients and controls, ie body mass index (BMI), smoking, previous CVD, blood pressure (BP), as well as previous fasting serum glucose and lipid levels (if available).

Patients and controls with BMI≥25 kg/m^2^ were considered overweight. Hypertension was defined as systolic BP ≥ 140 mm Hg and/or diastolic BP ≥ 90 mm Hg, or the use of antihypertensive drugs. Diabetes mellitus was defined as fasting glucose ≥7.0 mmol/L, or the use of antidiabetic medication. Hyperlipidemia was defined as total cholesterol ≥ 6.2 mmol/L, or low-density lipoprotein (LDL) cholesterol ≥ 3.61 mmol/L, or triglycerides≥1.7 mmol/L. Medical history of CVD included CAD/acute coronary syndrome, transient ischemic attacks, and ischemic stroke.

### Advanced ECG Data Collection and Analyses

A 12-lead ECG file was recorded for each participant for 5 min using the A-ECG device (Cardiax^®^, IMED Co Ltd, Budapest, Hungary). For each ECG file, at least 256 waveforms were acquired as previously described.^13^ A-ECG data analyzed included conventional parameters, derived vectorcardiographic parameters, parameters of QRS and T waveform complexity and parameters for the beat-to-beat variability of the R-to-R and QT intervals.^13^ A previously validated 9-parameter score, derived by multivariable logistical regression analysis, was calculated to determine the probability of having cardiac disease,^13^ and cut-off for positive results was defined as probability >0.50. In short, probability was calculated by using the 9-measure A-ECG score for “disease” derived from logistic regression, as the score itself generates a numerical result for each patient that will usually have some positive or negative numerical value. The score result itself is then transformed into a probability, between 0 and 1 for the given disease the score applies to, through the use of the constant “e” (ie, the “exponential function”, or Euler's number). Three other A-ECG scores were also used to evaluate the risk of left ventricular systolic dysfunction (LVSD), coronary artery disease/coronary microvascular disease (CAD/CMVD) and left ventricular hypertrophy/left ventricular electrical remodeling (LVH/LVER), respectively.^21^

### Overall Hemostatic Potential Assay

The overall hemostatic potential (OHP) assay was performed in a 96-well plate as previously described, with minor modifications.^22^ Briefly, 20 µL Phospholipid Reagent-TGT and 140 µL plasma were added to the wells, and coagulation was initiated by adding 100 µL Tris buffer containing Ca^2+^ and thrombin, with or without tissue-plasminogen activator (t-PA) to the wells. The final concentrations of each component were as follows: Phospholipid Reagent-TGT 0.02 mM, Ca^2+^ 13 mM, thrombin 0.04 U/mL, and t-PA 300 ng/mL The fibrin formation and fibrinolysis were monitored by measuring the absorbance (Abs) at λ=405 nm every minute for 90 min. Overall coagulation potential (OCP) was calculated as the area under the curve of fibrin formation, while the OHP as the area under the curve of fibrinolysis, and the overall fibrinolytic potential (OFP) as OFP=([OCP-OHP]/OCP) x 100%. The lag-phase was calculated as the time to the inflection point before Abs increase, and time to plateau as the time to the inflection point following Abs increase and up to when Abs reached a plateau. Max change of Abs equals to the Abs value at the time to plateau minus the Abs value at 0 min. Max velocity was calculated as the biggest change of Abs in two continuous minutes. The clot lysis time was calculated in the fibrinolysis curve (with addition of t-PA) and defined as the time interval between the midpoint of the Abs increase phase to the midpoint of Abs decrease phase.^23,24^

Patients that had received treatment with FVIII concentrate up to three days prior to blood sampling were excluded from the OHP analyses.

### EVs Analysis by Flow Cytometry

The EVs analyses were performed as previously described with minor modifications.^12^ Briefly, 20 µL plasma was incubated with 5 µL Annexin-V-FITC (PS + EVs), 5 µL mouse anti-human CD61-PE (PEVs), 5 µL mouse anti-human CD62P-APC and 15 µL PBS buffer. In a parallel tube, 20 µL plasma was incubated with 5 µL mouse anti-human CD51/61-FITC (EEVs), 5 µL mouse anti-human CD142-PE (TF + EVs), 5 µL mouse anti-human CD45-APC (LEVs) and 15 µL PBS buffer. The above two tubes for each sample were incubated for 20 min at room temperature in dark, and incubation was stopped by adding 500 µL PBS buffer. Isotype controls were used as negative controls. Tubes added with 1.25 µL 10% Triton X-100 were used to define the boundaries between EVs and the background debris or noises. The samples were analyzed by using the BD Canto II flow cytometer at low flow rate for 90 s, which enabled a 32 µL mixture in the tube being analyzed. The EV-size gate was defined for every run by using the Megamix SSC-Plus calibration beads (BioCytex, Marseille, France) and the gate size (0.3-1.0 µm) corresponds to size of microvesicles. Exosomes were excluded due to the inability of FACS Canto II and calibration beads to measure particles of their size. The concentration of EVs was equal to (Events count x 550 µL)/(20 µL x 32 µL). Calibration and gating strategy for identification of EVs and definition of populations is shown in Supplemental Materials, Figure 1.

Further details on the antibodies are provided in Supplemental Material, Table 1.

### Statistics

Graph Pad Prism (version 7.0) and IBM SPSS statistics (version 25.0) software were used to make statistical analysis. The data were presented as mean ± standard deviation (STD), and if not normally distributed, as median [interquartile range]. To compare continuous variables in two independent groups, t test or Mann-Whitney test were used as appropriate. To compare categorical variables, Pearson Chi-square test was used, or Fisher's exact test if more than 20% of the cells had expected values <5, or Chi-square test for trend if one of the variables had >2 levels. Correlations between parameters were analyzed using Spearman's rank correlation test. The statistical significance was defined as p < 0.05 for all tests.

## Results

### Cohorts

Forty-two patients with HA treated on-demand were included in the study. Of those, 24 patients had severe HA and 18 had non-severe HA (8 with mild and 10 with moderate HA). Data on risk factor profile for CAD and A-ECG were available for all patients. All patients had likewise left blood samples, but blood glucose and lipids were not measured in 16 patients. In the control group, 27 participants had complete data, including risk profile for CAD, A-ECG files, and blood samples. Risk factors for CAD or A-ECG files were not available for 10 participants.

Patients with severe HA were younger than those with non-severe HA (t-test) and had higher frequencies of spontaneous bleeding and bleeding after injury (Pearson Chi-Square test). However, the frequency of concentrate treatment was similar in the two groups (Pearson Chi-Square test). The clinical characteristics of the patients with HA are presented in Table 1.

**Table 1.** Characteristics of Patients with Hemophilia A Treated on-Demand.

Patient Characteristics, NO. (%)	Total Patients (n = 42)	Severe (n = 24, 57.1%)	Moderate-Mild (n = 18, 42.9%)
Age (years, mean ± SD)	42.3 ± 10.7	38.8 ± 8.6	46.9 ± 11.6
Age at first bleeding			
<6 years	35 (83.3%)	22 (91.7%)	13 (72.2%)
6–18 years	5 (11.9%)	2 (8.3%)	3 (16.7%)
>18 years	2 (4.8%)	0	2 (11.1%)
Age at diagnosis of HA			
<6 years	11 (26.2%)	8 (33.3%)	3 (16.7%)
6–18 years	11 (26.2%)	8 (33.3%)	3 (16.7%)
>18 years	20 (47.6%)	8 (33.3%)	12 (66.7%)
Frequency of spontaneous bleeding			
none	7 (16.7%)	0	7 (38.9%)
1–3 times/year	9 (21.4%)	4 (16.7%)	5 (27.8%)
3–10 times/year	6 (14.3%)	4 (16.7%)	2 (11.1%)
>10 times/year	20 (47.6%)	16 (66.7%)	4 (22.2%)
Frequency of bleeding after injury			
none	10 (23.8%)	3 (12.5%)	7 (38.9%)
1–3 times/year	25 (59.5%)	16 (66.7%)	9 (50.0%)
3–10 times/year	3 (7.1%)	1 (4.2%)	2 (11.1%)
>10 times/year	4 (9.5%)	4 (16.7%)	0
Frequency of replacement therapy			
none	2 (4.8%)	0	2 (11.1%)
1–3 times/year	9 (21.4%)	3 (12.5%)	6 (33.3%)
3–10 times/year	8 (19%)	7 (29.2%)	1 (5.6%)
>10 times/year	23 (54.8%)	14 (58.3%)	9 (50.0%)
Joint deformity and/or limited movement			
No	6 (14.3%)	1 (4.2%)	5 (27.8%)
Yes	36 (85.7%)	23 (95.8%)	13 (72.2%)
Family history of HA	27 (64.3%)	16 (66.7%)	11 (61.1%)
Hepatitis C virus infection	7 (16.7%)	5 (20.8%)	2 (11.1%)
Human immunodeficiency virus infection	0	0	0
History of surgery	15 (35.7%)	12 (50%)	3 (16.7%)
FVIII inhibitor	8 (19%)	7 (26.9%)	1 (6.3%)

### Risk Factors for CAD

Age was generally matched between the patients and controls (t-test). All the CAD risk factors studied were comparable between the groups (Mann-Whitney U test). In the patient group, 19% had hypertension, which is insignificantly higher compared to the control group (14.8%, p = 0.651) (Fisher's exact test). The levels of blood glucose and lipids did not show any statistical difference between the patients and controls (Fisher's exact test). Notably, one patient with non-severe HA, who had multiple risk factors for CAD (obesity, smoking, hypertension, diabetes mellitus and dyslipidemia), had experienced one ischemic stroke at the age of 39 years.

Risk factors for CAD in patients with HA and controls are shown in Table 2.

**Table 2.** Risk Factors for CAD in Patients with HA and the age-Matched Male Controls.

Risk Factors for CAD, Mean ± SD Or NO. (%)	Controls (n = 27)	Total Patients (n = 42)	Severe (n = 24)	Moderate-Mild (n = 18)
Age (years)	38.2 ± 10.3	42.2 ± 10.8	38.7 ± 8.8	46.9 ± 11.6*
Overweight	14 (51.9%)	15 (35.7%)	7(29.2%)	8 (44.4%)
Smoking	5 (18.5%)	17 (40.5%)	7(29.2%)	10 (55.6%)
Hypertension	4 (14.8%)	8 (19.0%)	3 (12.5%)	5 (27.8%)
Diabetes mellitus^#^	1 (3.7%)	2 (7.7%)	0	2 (16.7%)
Blood glucose (mM)^#^	4.9 ± 0.8	4.7 ± 0.9	4.3 ± 0.6	5.0 ± 1.1
Dyslipidemia^#^	9 (33.3%)	6 (23.1%)	2 (14.3%)	4 (33.3%)
T-CHO (mM)^#^	4.5 ± 0.8	4.2 ± 1.0	4.0 ± 1.3	4.3 ± 0.6
TG (mM)^#^	1.4 ± 0.8	1.3 ± 0.7	1.3 ± 0.7	1.4 ± 0.7
LDL (mM)^#^	3.0 ± 0.7	2.6 ± 1.2	2.3 ± 1.4	2.9 ± 0.6
CVD events	0	1 (2.4%)	0	1 (5.6%)

# Among the 42 patients, 26 (14 with severe HA, and 12 with non-severe HA) had blood glucose and lipids level measured. T-CHO, Total Cholesterol; TG, Triglycerides; LDL, Low Density Lipoprotein. * p < 0.05 as compared with the control group.

### A-ECG Analysis

**Figure 1.**
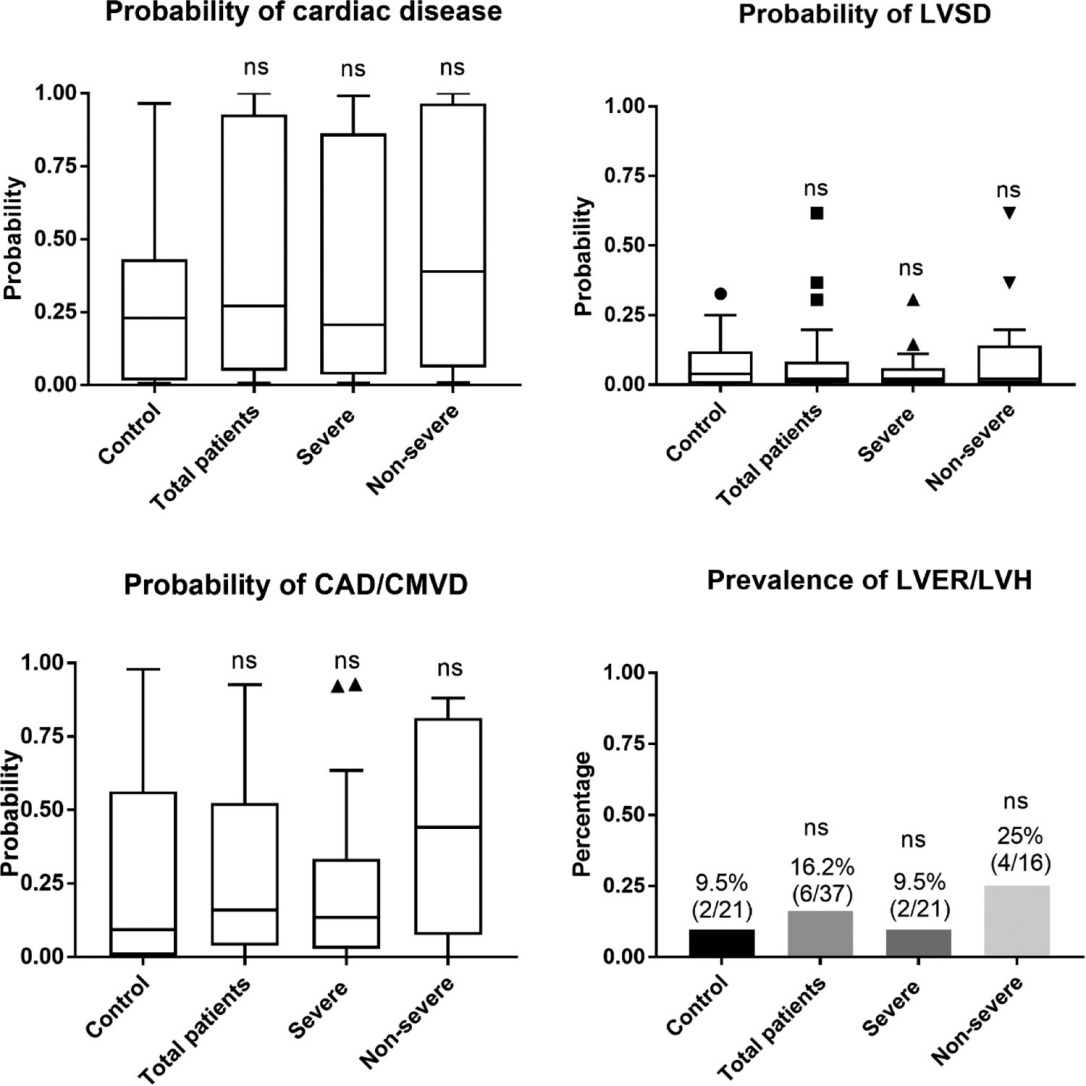
A-ECG results of the patients with HA (n = 42, n = 24 with severe and n = 18 with non- severe disease) and controls (n = 37). For calculation of probability, refer to the Methods section. Prevalence refers to the percentage within the given group (in this particular study) that had a probability for “LVER/LVH” of >0.5 after transformation of the logistic score results for LVER/LVH into probabilities, as described in Methods.

In total, A-ECG files from 37 patients and 21 controls could be analyzed. Five A-ECG files in the patient group and six in the control group were excluded due to the bad quality. Patients with HA had similar probability of cardiac disease compared to controls (median [interquartile range] 27.04 [4.87–92.61]% vs 22.89 [1.63–43.06]%, p = 0.204, Figure 1). More than one-third of the patients with HA (37.8%) were predicted to have overt or subclinical cardiac diseases as evaluated by Α-ECG, which was insignificantly higher compared to the control group (23.81%, p = 0.274). Patients with severe HA had insignificantly lower probability of having cardiac disease compared to the patients with non-severe HA (20.58 [3.58–86.16]% vs 38.80 [5.92–96.58]%, p = 0.294). Consistently, the predicted risk also tended to be lower in the group with severe HA (33.3% vs 43.8%, p = 0.517). The probabilities of LVSD, CAD/CMVD and/or LVH/LVER were similar between the patients and the controls (Figure 1). Mann-Whitney U test was used for those analyses.

### OHP Results

Patients with HA exhibited significantly impaired fibrin formation, as shown by the decreased OCP and OHP values, prolonged lag-phase and time to plateau and slower max velocity. Patients also exhibited shorter clot lysis time. The fibrin formation and fibrin stability against fibrinolysis were even more impaired in the patients with severe HA. The patients with non-severe HA had similar OCP, OHP and OFP values and clot lysis time as controls, however, the lag-phase and time to plateau were prolonged, and the max velocity was lower, as shown in Table 3. Mann-Whitney U test was used for those analyses.

**Table 3.** Results of the OHP Assay in the Study Participants.

OHP Assay Parameters	Controls (n = 37)	Total Patients (n = 36)^#^	Severe (n = 21)	Moderate-Mild (n = 15)
OCP	77.4 ± 14.3	41.4 ± 34.3*	21.1 ± 23.3*	69.8 ± 26.1
OHP	28.2 ± 11.4	14.3 ± 14.8*	5.3 ± 9.1*	27 ± 11.4
OFP	63.9 ± 11.3	69.5 ± 16.2	77 ± 15.9*	60 ± 10.8
Lag-phase	3.8 ± 1.7	22.3 ± 14.4*	26.7 ± 16.4*	16.1 ± 8*
Time to plateau	10.4 ± 5.2	65.7 ± 28.9*	82.2 ± 17.5*	42.5 ± 25.7*
Max change of Abs	0.8775 ± 0.1783	0.6878 ± 0.4255	0.4303 ± 0.3553*	1.0483 ± 0.183
Max velocity	0.5502 ± 0.1524	0.0955 ± 0.108*	0.0348 ± 0.0474*	0.1805 ± 0.1127*
Clot lysis time	32.3 ± 9.1	23.4 ± 12.7*	19 ± 12.5*	30.1 ± 10.2

# 6 patients were excluded because they had FVIII infusion within the past 3 days. *p ≤ 0.001 as compared to the control group.

### Analysis of EVs

**Figure 2.**
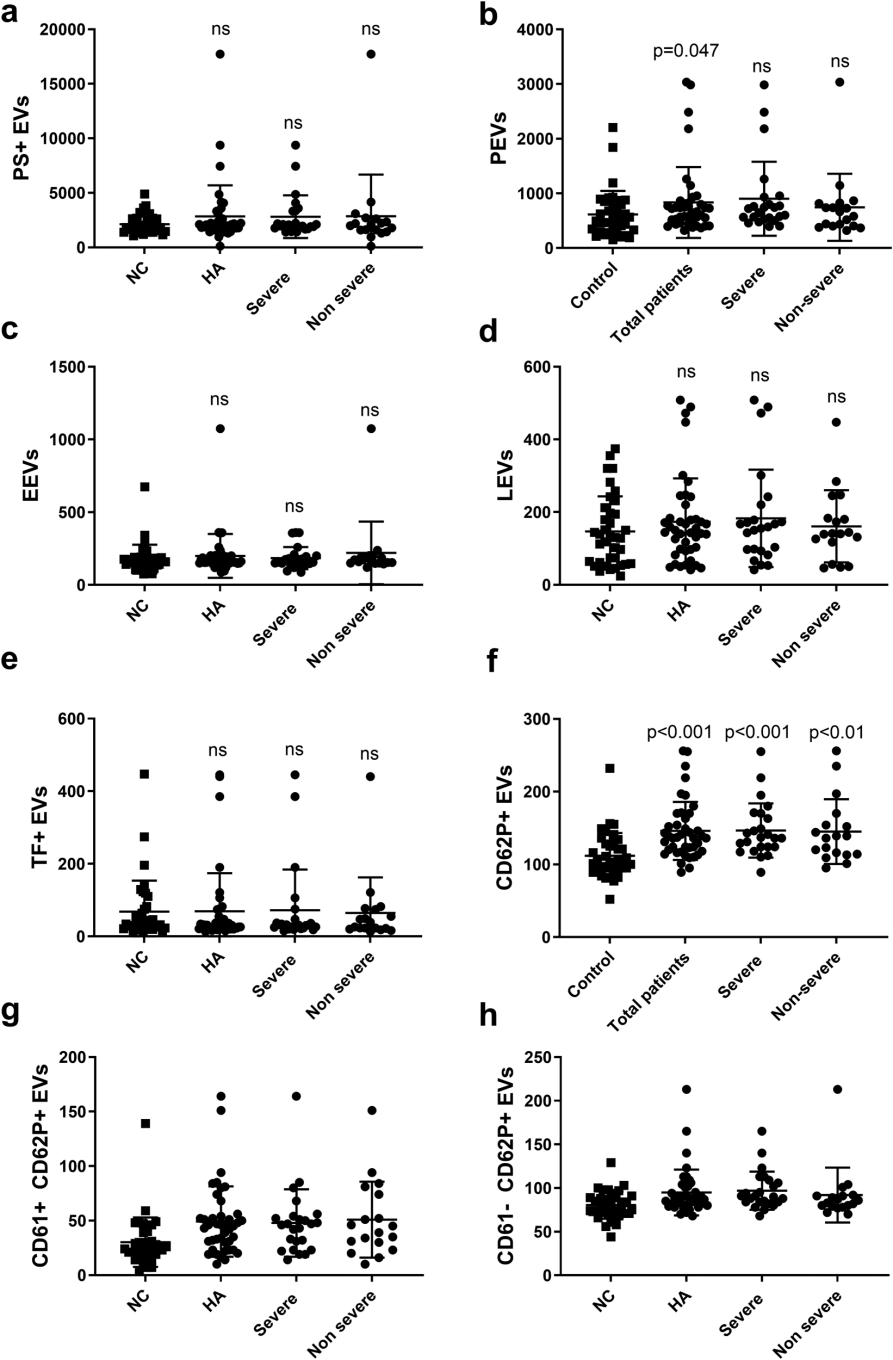
EVs profiles in patients with HA and in controls, ns (no significant difference) as compared to the control group (controls n = 37, total patients n = 42, severe n = 24, non-severe n = 18). The EVs were measured as events/μL.

Patients with HA had higher levels of PEVs (656.5 [472.0–826.8] vs 494.0 [310.0-823.5], p = 0.047) and CD62+ EVs (136.5 [117.8-164.8 vs 107.0 [93.0-128.5], p < 0.001) compared to controls (Figure 2). The levels of the other sub-types of EVs, including PS + EVs, EEVs, LEVs and TF + EVs, did not differ between the groups (Figure 2). To evaluate whether the severity of HA could influence the levels of EVs, we compared the levels of different subtypes of EVs between the patients with severe versus non-severe HA. No differences were found. Mann-Whitney U test was used for those analyses. The levels of CD62P + EVs were significantly correlated with the lag time (r = 0.366, p = 0.001), time to plateau (r = 0.427, p < 0.001) and OFP value (r = 0.337, p = 0.004), and inversely correlated with the OCP value (r = -0.266, p = 0.023), OHP value (r = -0.425, p < 0.001), max velocity (r = -0.479, p < 0.001) and the clot lysis time (r = -0.314, p = 0.007) (analyzed by Spearman's rank correlation test).

### Correlation of CAD Risks with OHP Parameters or EVs Levels

No correlations were found between CAD probabilities (as reflected by the A-ECG scores) and the OHP parameters, or the levels of EVs in patients with HA, as shown in Table 4 (analyzed by Spearman's rank correlation test).

**Table 4.** Correlation Analysis Between the CAD Risks, the Global Hemostatic status and the EVs Profiles of the Participants.

	The Probability of Having Cardiac Disease, P (r)
OCP	0.787 (−0.038)
OHP	0.364 (−0.127)
OFP	0.821 (0.033)
Lag-phase	0.154 (0.199)
Time to plateau	0.239 (0.165)
Max change of Abs	0.537 (0.087)
Max velocity	0.458 (−0.104)
Clot lysis time	0.793 (−0.037)
PS + EVs	0.503 (0.090)
PEVs +	0.742 (0.044)
EEVs +	0.473 (0.096)
LEV +	0.599 (0.071)
TF + EVs	0.693 (−0.053)
CD62P +	0.280 (0.144)

r, Spearman's correlation coefficient.

## Discussion

In this study, patients with HA treated on-demand and age-matched male controls had similar probabilities of cardiac diseases as evaluated by an A-ECG derived score. Patients with HA had impaired fibrin formation and fibrin stability against fibrinolysis, as well as elevated levels of PEVs and CD62P + EVs. The correlation between the levels of CD62P + EVs and deficient fibrin clot formation, suggests that coagulation impairment might lead to enhanced platelet activation in patients with HA.

We found that patients with HA had similar prevalence of hypertension as age-matched male controls, which is consistent with some,^8,25^ but not all^26,27^ previous studies. The results on the comparisons for the other risk factors for CAD were similar. This is indicative of a similar prevalence of hypertension, diabetes mellitus and hyperlipidemia in both controls and patients with HA and underlines the need for vigorous screening and follow-up to ensure adequate and timely intervention in order to avoid therapeutic dilemmas, ie having to use platelet inhibitors following a cardiovascular event.

By using the A-ECG method, we found that the probability of having cardiac disease tended to be higher in patients with HA compared to the controls, although there was no significant difference. Interestingly, in a previous retrospective study on a Swedish cohort, we found that patients with HA had more overt or subclinical CAD, as evaluated by the same A-ECG technique as in this study, compared to age-matched male controls.^13^ It is unclear whether this estimated higher CAD probability is associated with the fact that the patients with severe HA are routinely treated prophylactically in Sweden and likewise whether CAD is increased in elderly HA patients treated regularly. Sood et al reported that the prevalence of CVD was similar in patients with HA treated prophylactically and those treated on-demand.^26^ However, they focused only on clinical CVD, while the A-ECG technique can detect both clinical and subclinical changes suggestive of CAD. The A-ECG technique has been validated as more sensitive to identify certain cardiac diseases compared to standard ECG.^13–16^ Subclinical CVD in patients with HA could be evaluated by other techniques, such as echocardiography which has shown increased septal thickness and diastolic dysfunction.^28^ Even though there is considerable potential in the usage of this A-ECG method, it has not been evaluated in a broader clinical setting and recommendations on clinical use cannot be made based on current knowledge alone. However, the results can be part of the clinical evaluation and possibly indicate the need for a more thorough assessment by utilizing standardized and established imaging methods.

In our study, patients with HA had impaired fibrin formation and faster fibrinolysis compared to controls, as previously shown.^12^ Patients with non-severe HA had relatively normal global hemostasis, with similar OCP and OHP values as the control group. However, the lag-phase and time to plateau were prolonged, and the max velocity of fibrin formation was lower in the patients with non-severe HA compared to controls. Although increased clot formation has been found in patients with CVD,^29^ no correlation between the probability of cardiac disease and the OHP assay parameters was found.

The patients with HA had elevated levels of PEVs, which is consistent with a previous study.^30^ However, the levels of LEVs and EEVs were similar between patients and controls. This discrepancy might be attributed to the differences in the cohorts. The cohort in the previous study consisted of previously untreated children, while our study recruited patients older than 30 years treated on-demand.^30^ We found that the number of CD62P + EVs were higher in patients than in controls, suggesting a higher degree of platelet activation. One earlier study reported increased CD62P exposure in platelets of patients with HA at baseline, suggesting a pre-activated state.^31^ However, the baseline activation of platelets did not correlate with bleeding severity.^32^ The levels of CD62P + EVs were similar between the patients with severe versus non-severe HA in our cohort. Interestingly, we found that the level of CD62P + EVs was correlated with the impairment of fibrin formation, suggesting that a hypocoagulable state might be the reason for increased platelet activation. While impaired coagulation may contribute to enhanced platelet activation, it is also plausible that it leads to diminished incorporation of EVs into the hemostatic plug and delayed clearance from circulation. This could alter both the local and systemic roles of platelet EVs in coagulation and inflammation. The interplay between EV dynamics, platelet activation, and coagulation impairment is multifactorial and context-dependent, underscoring the need for nuanced interpretation and further investigation into their complex biological role.

Previous studies have reported elevated EVs, particularly EEVs, in patients with CVD.^11,33^ EEVs are released from endothelial cells upon activation or apoptosis and serve as biomarkers of endothelial dysfunction. In the current study, we did not find any correlation between the EV levels and the risk of cardiac diseases as evaluated by the A-ECG. The levels of EEVs were similar between the patients and controls, suggesting that patients with HA treated on-demand are not protected from endothelial dysfunction. Some other studies have reported impaired endothelial function in patients with HA.^34,35^ Patients with HA had increased levels of several soluble biomarkers, ie the soluble intercellular adhesion molecule-1 and interleukin-6 in serum, suggesting decreased endothelial function.^34^ Moreover, Sartori et al reported a higher prevalence of endothelial dysfunction in patients with hemophilia compared to controls by measuring flow-mediated dilation using ultrasound.^35^

The main strength of our study is that it focused on patients with HA treated on-demand. If a deficiency of FVIII could protect against ischemic heart diseases, it would be more overt in patients treated on-demand, since prophylaxis could weaken this protective effect. A major limitation of this study was the small number of participants as well as the missing values, which might have hampered the statistical power. However, we found that patients with HA had similar prevalence of traditional risk factors for CAD and showed a non-significant tendency towards a slightly higher probability for cardiac diseases. Our data suggest that in patients with HA treated on-demand, the risk of developing CAD should be recognized as the patients become older than 30 years old, namely the age when atherosclerosis begins.^36^ Another limitation of the study is the fact that A-ECG is not employed in clinical risk stratification and has therefore not been evaluated in this setting. The results of the study however serve as a starting point for further prospective studies with follow-up, rather than to introduce a tool for immediate clinical use. It should also be noted that the patients in this cohort have a considerable bleeding phenotype, which can lead to hemostatic activation and potentially indicating deviation from the management recommendations. Since this is a real-world, unselected cohort, all treatment decisions were made by the physician responsible for the patient, independently of the authors’ insight.

As noted earlier, we hypothesized that patients with HA treated prophylactically could have higher CAD risk, as evaluated by an A-ECG score, compared to those treated on-demand due to the formers’ improved coagulation capacity. We are currently conducting a cross-sectional study with patients treated prophylactically, in order to evaluate their risks of developing CAD and the correlations between said risks, the levels of EVs and the results of global hemostatic tests.

In conclusion, we found that patients with HA treated on-demand do not have fewer abnormalities in A-ECG compared to controls. In the context of this study and in accordance to how A-ECG has been utilized, this could possibly indicate that they do not appear to be protected from the risk of developing cardiac diseases. The higher levels of PEVss and CD62+ EVs could indicate a pre-activation of platelets in patients with HA, which might be a reaction to their hypocoagulable status. However, the lack of definitive correlations between laboratory and electrocardiographic findings, as well as the missing values and small cohort limits the conclusions that can be drawn. Our study lays the ground for larger, preferably multi-centre studies, where the larger number of participants and probably fewer missing values could contribute to more robust conclusions and clinical implications.

## Supplemental Material

sj-pdf-1-cat-10.1177_10760296261432444 - Supplemental material for Subclinical Cardiac Diseases and the Role of Extracellular Vesicles in Patients with Hemophilia A Treated on-Demand Supplemental material, sj-pdf-1-cat-10.1177_10760296261432444 for Subclinical Cardiac Diseases and the Role of Extracellular Vesicles in Patients with Hemophilia A Treated on-Demand by Yanan Zong, Maren Maanja, Todd T. Schlegel, Martin Ugander, Jovan Antovic, Apostolos Taxiarchis, Roza Chaireti and Xiangdong Kong in Clinical and Applied Thrombosis/Hemostasis
