# Supplementary material for: Subclinical Cardiac Diseases and the Role of Extracellular Vesicles in Patients with Hemophilia A Treated on-Demand
Source: Clin Appl Thromb Hemost. 2026 Mar 9;32:10760296261432444. doi: 10.1177/10760296261432444 (PMC12972547; doi:10.1177/10760296261432444)
Supplement: sj-pdf-1-cat-10.1177_10760296261432444 - Supplemental material for Subclinical Cardiac Diseases and the Role of Extracellular Vesicles in Patients with Hemophilia A Treated on-Demand [file sj-pdf-1-cat-10.1177_10760296261432444.pdf]

Supplementary Material

Subclinical cardiac diseases and the role of extracellular vesicles in patients with hemophilia A treated on-demand.

Yanan Zong, Maren Maanja, Todd Schlegel, Martin Ugander, Jovan P. Antovic, Apostolos Taxiarchis, Roza Chaireti*, Xiangdong Kong

*** Correspondence:** Corresponding Author: roza.chaireti@ki.se

Supplementary Figures

Antibodies	Source	Identifier	Dilution
FITC Annexin V	BD Biosciences	556420	5 ul/test
PE Mouse Anti-Human CD61	BD Biosciences	555754	5 ul/test
APC Mouse Anti-Human CD62P	BD Biosciences	550888	5 ul/test
FITC Mouse Anti-Human CD51/CD61	BD Biosciences	555505	5 ul/test
PE Mouse anti-human CD142	BD Biosciences	550312	5 ul/test
APC Mouse Anti-Human CD45	BD Biosciences	555485	5 ul/test
FITC Mouse IgG1, κ Isotype Control	BD Biosciences	349041	5 ul/test
PE Mouse IgG1, κ Isotype Control	BD Biosciences	349043	5 ul/test
APC Mouse IgG1 κ Isotype Control	BD Biosciences	550854	5 ul/test

Supplementary Table 1. List of antibodies.

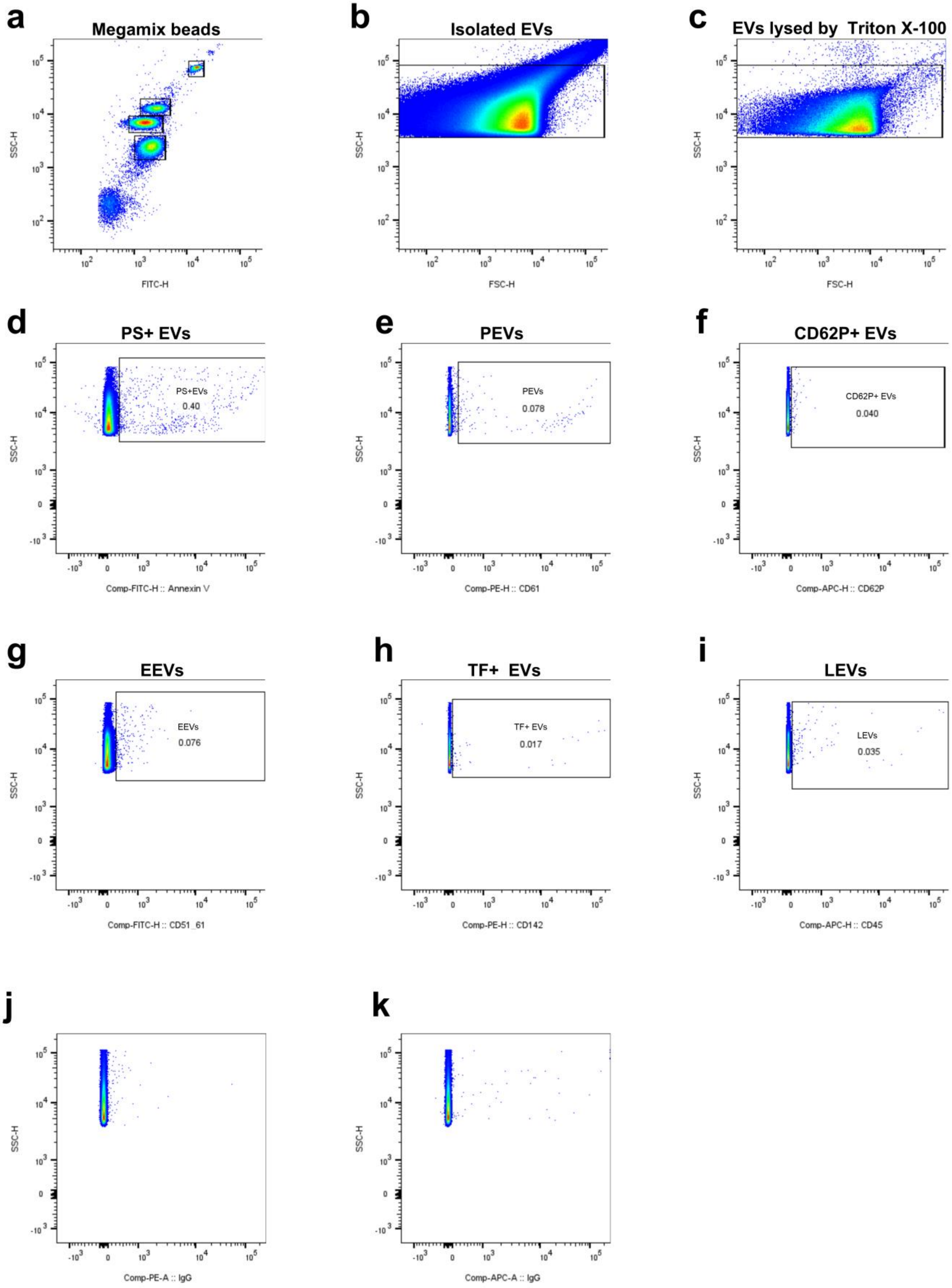
a
Megamix beads
SSC-H
FITC-H
b
Isolated EVs
SSC-H
FSC-H
c
EVs lysed by Triton X-100
SSC-H
FSC-H
d
PS+ EVs
PS+EVs
0.40
SSC-H
Comp-FITC-H :: Annexin V
e
PEVs
PEVs
0.078
SSC-H
Comp-PE-H :: CD61
f
CD62P+ EVs
CD62P+ EVs
0.040
SSC-H
Comp-APC-H :: CD62P
g
EEVs
EEVs
0.076
SSC-H
Comp-FITC-H :: CD51_61
h
TF+ EVs
TF+ EVs
0.017
SSC-H
Comp-PE-H :: CD142
i
LEVs
LEVs
0.035
SSC-H
Comp-APC-H :: CD45
j
SSC-H
Comp-PE-A :: IgG
k
SSC-H
Comp-APC-A :: IgG

Supplementary Figure 1. Calibration and gating strategy for detection of EVs. (a) Calibration with 0.16–0.5 µm standard Megamix SSC-Plus beads (corresponding to 0.3–1.0 µm Megamix FSC beads). This size corresponds to the size of microvesicles. (b) events in the gate represent the size range of isolated EVs. (c) number of EVs (number of events) in the gate decreases due to lysis with Triton X-100. (d-i) Gating strategy for determination of: (d) Annexin-V-FITC (PS+) EVs; (e) CD61 (PEVs) EVs: (f) CD62-P+ EVs; (g) CD51/61 (EEVs) EVs; (h) CD142 (TF+) EVs; (i) CD45 (LEVs) EVs; (j-k) Isotype controls (IgG)
